# Natural history of angiomyolipoma in lymphangioleiomyomatosis: implications for screening and surveillance

**DOI:** 10.1186/s13023-014-0151-3

**Published:** 2014-10-03

**Authors:** Zhao W Yeoh, Vidya Navaratnam, Rupesh Bhatt, Ian McCafferty, Richard B Hubbard, Simon R Johnson

**Affiliations:** Divisions of Respiratory Medicine and Respiratory Research Unit, School of Medicine, University of Nottingham, D Floor, South Block. Queens Medical Centre, Nottingham, NG7 2UH UK; Epidemiology and Public Health, School of Medicine, University of Nottingham, Nottingham, UK; School of Medicine, University of Nottingham, Renal tumour service, University Hospital Birmingham, Nottingham, UK; National Centre for Lymphangioleiomyomatosis, Nottingham University Hospitals NHS Trust, Nottingham, UK

**Keywords:** Lymphangioleiomyomatosis, Tuberous sclerosis, Kidney disease, Natural history

## Abstract

**Background:**

LAM is a rare disease of women categorised by lung cysts and lymphatic abnormalities. The disease occurs sporadically or associated with Tuberous Sclerosis Complex (TSC-LAM). Angiomyolipoma, a benign tumour, prone to haemorrhage, occurs mostly in the kidneys in many of these patients. Treatment guidelines exist for angiomyolipoma in patients with TSC but the natural history of angiomyolipoma in sporadic LAM has not been studied.

**Aims:**

To document the natural history of angiomyolipoma in a national cohort of patients with sporadic LAM to inform tumour screening and surveillance protocols.

**Methods:**

Demographic data, clinical features, lung function and tumour size were obtained from clinical records of patients attending the National Centre for LAM in Nottingham, UK.

**Results:**

122 patients with definite or probable LAM by European Respiratory Society criteria were identified. One hundred and seven had sporadic LAM, of which 53 (50%) had at least one angiomyolipoma. In patients with sporadic LAM presentation of angiomyolipoma preceded or followed onset of lung symptoms by up to 11 and 38 years respectively. Mean tumour size was 28 mm (range 5-140 mm) at presentation and growth was 1.8 mm/yr (95% C.I. 0.42-3.82) thereafter. Eleven patients with sporadic LAM had had a nephrectomy due to angiomyolipoma bleeding. The need for intervention did not differ between those with TSC-LAM and sporadic LAM.

**Conclusions:**

Patients with LAM have a high prevalence of symptomatic angiomyolipoma which can present at any time. Angiomyolipoma in sporadic-LAM have a similar risk of bleeding to those with TSC. All patients should be screened for angiomyolipoma at diagnosis of lung disease by MRI scanning and the tumours require continuous monitoring.

## Introduction

Lymphangioleiomyomatosis (LAM) is a rare systemic disease, almost exclusively affecting women with a prevalence of 5–10/million women [1]. LAM causes lung cysts and lymphatic abnormalities leading to recurrent pneumothorax, respiratory impairment and chylous collections [2]. LAM occurs both sporadically and in patients with tuberous sclerosis complex (TSC). Angiomyolipoma, a benign tumour, is present in up to half of patients with sporadic LAM [3,4] and nearly all patients with TSC-LAM [5,6].

The majority of angiomyolipomas do not cause symptoms although larger tumours are at risk of bleeding. Failure to identify and prophylactically treat larger tumours with either selective embolisation or nephron sparing surgery can result in retroperitoneal haemorrhage and sometimes a nephrectomy [7]. More recently, pharmacologic inhibition of the kinase mTOR has been shown to reduce angiomyolipoma volume in patients with TSC and sporadic LAM [8-10] and mTOR inhibitors are now recommended for treatment of angiomyolipoma in TSC [11].

Guidelines for management of angiomyolipoma are based on case series [12,13] and suggest that angiomyolipoma in patients with TSC grow more rapidly and are more prone to complications than angiomyolipoma in non-TSC patients [7]. However data on the natural history of angiomyolipoma in sporadic LAM are very sparse and it is not clear if angiomyolipoma in these patients really are less prone to haemorrhage and should be treated differently from those with TSC. We have examined the clinical characteristics, growth rate and complications of angiomyolipoma in women with sporadic-LAM to optimise screening protocols for these patients.

## Methods

### Patients

**Figure 1:**
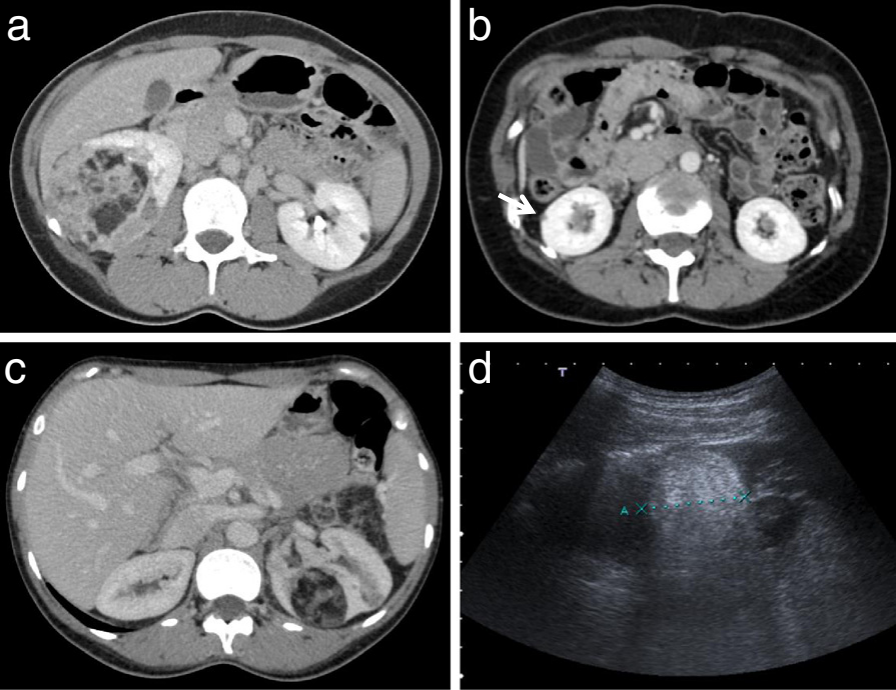
**Imaging appearances of angiomyolipomas. (a)** Characteristic CT appearance of large right renal angiomyolipoma showing a heterogenous lesion with areas of fat density and a small lesion in the contralateral kidney. **(b)** Tiny exophytic cortical angiomyolipoma in the right kidney assigned a measurement of 5 mm (arrow). **(c)** & **(d)** CT and ultrasound appearances respectively of the same 40 mm angiomyolipoma.

Patients were recruited from the National Centre for LAM in Nottingham UK, a referral centre providing comprehensive care for both sporadic and TSC-LAM. All patients were over the age of 18 years and had either definite or probable LAM according to European Respiratory Society (ERS) criteria [14]; those with possible LAM were excluded. TSC was diagnosed according to current criteria [15]. All patients had a clinical examination looking for signs of TSC including a dermatologic examination with a Woods light. In keeping with the ERS LAM guidelines; where TSC could not be excluded clinically, patients were evaluated by a clinical geneticist. Ethical approval was obtained from the Trent Multi-Centre Research Ethics Committee (NRES 07/H0403/165 and NRES 05/Q2403/187) and all patients provided informed consent. Patient histories were taken in a standardised format for clinical purposes and baseline assessment included screening for TSC as recommended in the ERS LAM guidelines [14]. Lung function tests were measured according to ERS/British Thoracic Society standards as part of clinical care [16]. Lymphatic involvement was evaluated at baseline and defined as the presence of chylous collections in the abdomen or thorax, abdominal or pelvic lymphadenopathy, diffuse lymphatic enlargement or lymyphangioleiomyomas visible by CT scanning. Renal history and imaging was obtained from both the referring centre and investigations performed at the National LAM Centre. Angiomyolipoma imaging was performed for routine clinical care according to the LAM Centre angiomyolipoma protocol where patients have cross sectional imaging of their renal tract, at or as soon after diagnosis as practical, and for those with angiomyolipoma, a measurement of tumour size at yearly intervals thereafter. Tumour size measurements obtained prior to attending the LAM Centre were performed at the referring clinician’s discretion. Tumour size was expressed as the longest diameter of angiomyolipomas measured by either ultra-sound (US), computerised tomography (CT) or magnetic resonance imaging (MRI) as previously described (Figure 1) [9]. Measurements were performed by either the reporting radiologist, or a member of the study team (SJ). Unquantifiable small tumours were assigned a measurement of 5 mm. Tumour measurements post angiomyolipoma surgery, post embolization or when using mTOR inhibitors were excluded from the analysis of tumour growth.

### Analysis

Groups were compared using linear regression, chi square or t-tests. Tumour growth rate (mm/yr) was defined as the slope of a regression line fitted to tumour measurements for patients who had repeated imaging. Tumour size categories were compared by a 1 way analysis of variance (ANOVA). Time to intervention was analysed by Kaplan-Meier analysis. Intervention was defined as a composite endpoint comprising action taken in response to spontaneous tumour bleeding, use of an mTOR inhibitor for angiomyolipoma, or referral to an interventional service at the discretion of the supervising clinician. Calculations were performed using Graphpad Prism version 6.00, (GraphPad Software, San Diego California USA) and Stata v11 (Texas)).

## Results

### Patient cohort

One hundred and twenty two patients with definite or probable LAM by ERS criteria were identified [14]. One hundred and seven patients had sporadic LAM and 15 TSC-LAM. Of those with sporadic LAM, the mean age at diagnosis with respiratory disease was 35.3 years (SD 10.4). The mean age at the time of the study was 50 years (SD 11.6). Presentation of respiratory disease was due to shortness of breath in 53%, pneumothorax in 28%, cough in 4%, and haemoptysis in 3%. The remaining 12% presented with either extra-pulmonary manifestations or were found to have LAM after investigation for unrelated problems.

### Presentation with angiomyolipoma

Of the 107 patients with sporadic LAM, 53 (50%) had, or had had, at least one renal angiomyolipoma. Two patients with renal angiomyolipoma had co-existent hepatic angiomyolipomas. Hepatic angiomyolipomas were not included in the analysis. The mean age at discovery of angiomyolipoma was 39.1 years (SD 13.2). Nineteen out of 53 (36%) of those with angiomyolipoma presented due to symptoms. In 11 patients this was abdominal or flank pain, in three haematuria, in three palpable mass and in two abdominal bloating. Of these patients presenting with angiomyolipoma symptoms, 11 were treated by nephrectomy, including one with renal bleeding during pregnancy. The median age at nephrectomy was 23 (range 13–49). Nine of these 11 had bilateral angiomyolipoma. The remaining 64% of angiomyolipomas were detected by screening after the diagnosis of lung disease (Table 1).

**Table 1:** **Characteristics of patients with or without angiomyolipoma**

	**Sporadic-LAM**			**TSC-LAM**
	**Angiomyolipoma**			
	**Present**	**Absent**	**p value**	
**Number of patients**	53	54		15
**Mean age at respiratory presentation** ^*^ **(years)**	32.3	37	0.18	31.8
**Mean disease duration** ^*^ **(years)**	14.4	15.2	0.21	10.5
**Ever had pneumothorax** ^**#**^ **(%)**	16 (32)	13 (24)	0.37	40
**Lymphatic disease detected** ^**#**^ **(%)**	6 (11)	10 (19)	0.036	13
**Ever been pregnant** ^**#**^ **(%)**	29 (55)	24 (44)	0.07	33
**Mean baseline FEV**_1_^*^ **(% predicted)**	65.7	71.4	0.37	64
**Mean baseline TL**_CO_^*^ **(% predicted)**	57.7	59.3	0.79	47

p = angiomyolipoma present vs. absent for patients with sporadic LAM.

Angiomyolipoma was present in all patients with TSC-LAM.

^*^p value from *t* test.

^#^p value from chi squared test.

**Figure 2:**
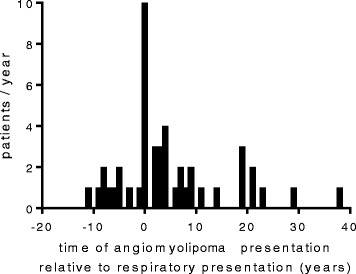
**Angiomyolipoma may present at any point in the disease course.** Frequency distribution of angiomyolipoma presentation relative to presentation with respiratory disease (respiratory diagnosis is at 0 years). Detection of angiomyolipoma may have been due to symptomatic disease or screening.

In 10 patients detection of angiomyolipoma preceded the diagnosis of lung disease by up to 11 years. In 10, the diagnosis of lung disease was made at the time of renal presentation but in the remainder, angiomyolipoma was detected up to 38 years after the onset of respiratory symptoms (Figure 2). Reliable tumour measurements for patients treated with nephrectomy at diagnosis were not generally available and could not be included in tumour size data. At presentation, the mean tumour size for patients with sporadic LAM was 28 mm (SD 12.6, Table 2). The presence of angiomyolipoma was not associated with duration of disease (p = 0.72) or the degree of loss of FEV_1_ (p = 0.37). Patients with angiomyolipoma were less likely to have lymphatic involvement than those without angiomyolipoma (p = 0.036) but other clinical features did not differ significantly between groups (Table 1).

**Table 2:** **Comparison of angiomyolipoma characteristics in patients with sporadic and TSC-LAM**

	**Sporadic-LAM**	**TSC-LAM**	**p value**
**Total number of patients**	**107**	**15**	
**Patients with angiomyolipoma** ^**#**^ **(%)**	50	100	<0.0001
**Mean age at renal presentation** ^*^ **(years)**	39	38	NS
**Mean tumour size at presentation** ^*^ **(mm)**	29	61	0.006
**Bilateral tumours** ^**#**^ **(%** ^**†**^ **)**	40	84	0.004
**Any renal tumour symptoms** ^**#**^ **(%** ^**†**^ **)**	49	40	NS
**Nephrectomy** ^**#**^ **(%** ^**†**^ **)**	21	13	NS

^*^p value from *t* test. NS = not significant.

^#^p value from chi squared test.

^†^percentage of those with angiomyolipoma.

Angiomyolipoma were present in all patients with TSC-LAM and significantly more common than in those with sporadic-LAM (p < 0.0001, Table 2). Compared with TSC-LAM, patients with sporadic LAM tended to have smaller tumours which were less often bilateral. However there was no significant difference in the incidence of bleeding or the need for intervention between the two groups (Table 2).

### Natural history of angiomyolipoma

**Figure 3:**
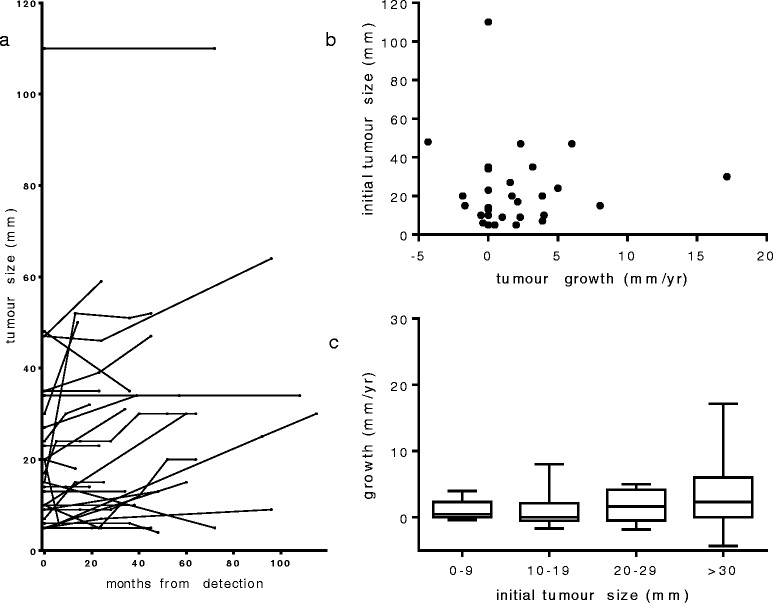
**Angiomyolipoma growth and tumour size. (a)** Serial tumour measurements of longest diameter for 31 angiomyolipomas where more than one measurement was available in patients with untreated angiomyolipomas. **(b)** Tumour size at detection and subsequent tumour growth. **(c)** Angiomyolipoma for patients with sporadic LAM categorised according to size at detection. Box plots show mean, interquartile range and range for tumour growth by size category. There is no difference in mean values between tumour size groups (p = 0.36 by one-way ANOVA) but growth of >10 mm/year was only observed in tumours greater than 30 mm.

More than one tumour measurement was available for 31 tumours from 26 patients with sporadic LAM. The mean duration of follow up was 3.8 years (SD 2.25). Seventeen (55%) tumours increased in size, nine (29%) did not change and five (16%) appeared to reduce in size (Figure 3a). The mean rate of growth for angiomyolipoma in sporadic LAM was 1.8 mm/year (95% confidence interval 0.42-3.82). Although there was no overall relationship between tumour size and growth, there was a trend toward larger tumours growing more rapidly (Figure 3b) and only those 30 mm or larger grew by more than 10 mm/yr (Figure 3c).

**Figure 4:**
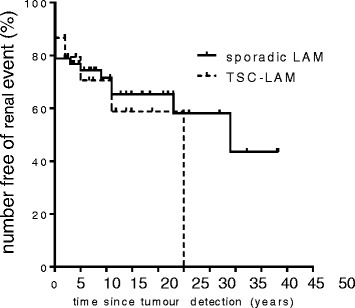
**Time to need for intervention for angiomyolipoma.** Kaplan-Meier curves show the time to a significant renal event (categorised as spontaneous tumour bleeding, use of an mTOR inhibitor, or referral to an interventional service at the discretion of the supervising clinician) for patients with sporadic and TSC-LAM. There was no significant difference in the need for intervention between patients with sporadic and TSC-LAM.

The median time from identification of angiomyolipoma to a clinically significant renal event, defined as referral for embolization or surgery, mTOR inhibitor treatment, bleeding or other tumour symptom, was 34 years for patients with sporadic LAM and 25 years for TSC-LAM (range 0–43) (Figure 4). There was no significant difference in clinically significant renal events between those with sporadic or TSC-LAM.

## Discussion

To our knowledge this is the first study to examine the natural history of angiomyolipoma in a large cohort of patients with sporadic LAM. Our aim was to understand the growth and incidence of bleeding in patients with sporadic LAM to develop better screening protocols for these tumours. Our findings show that angiomyolipomas are present in half of patients with sporadic LAM, can present at any time during the clinical course and are prone to growth and haemorrhage. Tumour presence cannot be predicted by the extent of the lung disease or other clinical features. This is important, as although patients with LAM are generally under regular follow up for lung disease, renal angiomyolipomas may be neglected and present with bleeding resulting in loss of renal function even in those with mild lung disease.

Previous series of angiomyolipoma may have underestimated the rates of TSC and LAM, particularly those with milder clinical features [17]. For this reason it has not been possible to address whether patients with non-TSC related angiomyolipoma are truly at lower risk of complications and should be treated differently from those with TSC. In contrast to previous studies, our findings suggest that patients with sporadic LAM and angiomyolipoma have a similar risk of complications to both historical series of those with TSC [7,18,19] and with the small number of TSC-LAM patients within our cohort.

Eleven of 53 patients had a nephrectomy due to haemorrhage from an angiomyolipoma. Nephrectomy due to bleeding generally preceded the diagnosis of lung disease and the histological type of renal tumour was unknown at presentation. These patients had a mean age at nephrectomy of 23 years whereas the mean age at renal presentation of the cohort as a whole was 39 years and it is likely that these patients had more aggressive and rapidly growing tumours.

Despite this being one of the largest studies of the behaviour of angiomyolipoma, and the first to document growth over time, our findings need to be interpreted with some caution as some of these data are retrospective and some patients had had treatment for symptomatic tumours prior to enrolment. As these treated tumours were probably larger and more aggressive, overall growth rates and the incidence of complications may be underestimated. Furthermore, some outcome data are based upon surrogate endpoints such as referral for an intervention, which may have been influenced by factors other than tumour size alone. In the cohort studied, more patients with sporadic LAM were referred electively for consideration of an intervention than those with TSC. Additionally, data were collected using different imaging modalities and protocols which may impact upon the growth data, particularly where smaller lesions may be of similar size to the slice intervals used in cross sectional imaging. Further, as ultrasound is not as accurate for size measurements as CT or MRI and also less reliable at identifying the fat poor components of angiomyolipomas we attributed a value of 5 mm to all tumours with a longest diameter of 5 mm or less, and these issues may explain why some of the tumours appeared to shrink over time.

The study also examines only tumour size and does not account for the presence of vascular aneurysms which are also associated with bleeding [18]. Analysing the vascular anatomy of these tumours may be required to improve stratification of bleeding risk and can be done with both contrast CT and MRI.

### Implications for clinical practice

Guidelines for patients with TSC have been recently published which recommend MRI of the abdomen to assess the progression of angiomyolipoma (and renal cystic disease) every 1–3 years throughout the lifetime of the patient. Intervention is recommended for tumours larger than three cm which is a more aggressive approach than that previously taken [11]. Patients with sporadic LAM are less prone to loss of renal function than those with TSC who commonly have multiple angiomyolipomas and may also have polycystic kidney disease. However, our findings suggest that patients with sporadic LAM are at significant risk of bleeding from angiomyolipoma, that these tumours may present at any point in the disease course, and grow unpredictably at a rate independent of progression of the lung disease. Although, from somewhat limited data, our findings suggest that only tumours greater than 30 mm are likely to grow in excess of 10 mm/yr and reach a size of 40–45 mm before further imaging and thus be at greater risk of haemorrhage. We would therefore suggest that all patients are screened for the presence of angiomyolipoma at diagnosis by MRI. For those with tumours less than 10 mm, imaging should be carried out every two years, for tumours between 11 and 30 mm in diameter, at 12 monthly intervals. For tumours greater than 30 mm, repeat imaging should be performed at 6 monthly intervals or referred for consideration of an intervention at that point. All patients with angiomyolipoma should be warned of the symptoms of bleeding and imaging should be performed urgently in the presence of new symptoms possibly attributable to angiomyolipoma.

In summary, patients with sporadic LAM have a high prevalence of symptomatic angiomyolipoma which can present at any time during the course of the disease. Angiomyolipoma in sporadic LAM have a similar risk of bleeding to those with TSC and require continuous monitoring. Prospective evaluation of large cohorts of patients with LAM in the developing specialist services is required to improve understanding of the natural history, optimal screening protocols and risk stratification of these tumours.

### Notation of prior abstract publication/presentation

Some data from the study were presented at the British Thoracic Society Winter Meeting, 2013.

## Abbreviations

ANOVA: Analysis of variance
CT: Computerised tomography
ERS: European Respiratory Society
LAM: Lymphangioleiomyomatosis
MRI: Magnetic resonance imaging
mTOR: mammalian target of rapamycin
SD: Standard deviation
TSC: Tuberous Sclerosis Complex
US: Ultra-sound
